# Supplementary figures and images for: Assessing the relationship between operationally defined zero-dose communities and access to selected primary healthcare services for children and pregnant women in emergency settings
Source: PLoS One. 2023 Feb 16;18(2):e0281764. doi: 10.1371/journal.pone.0281764 (PMC9934415; doi:10.1371/journal.pone.0281764)

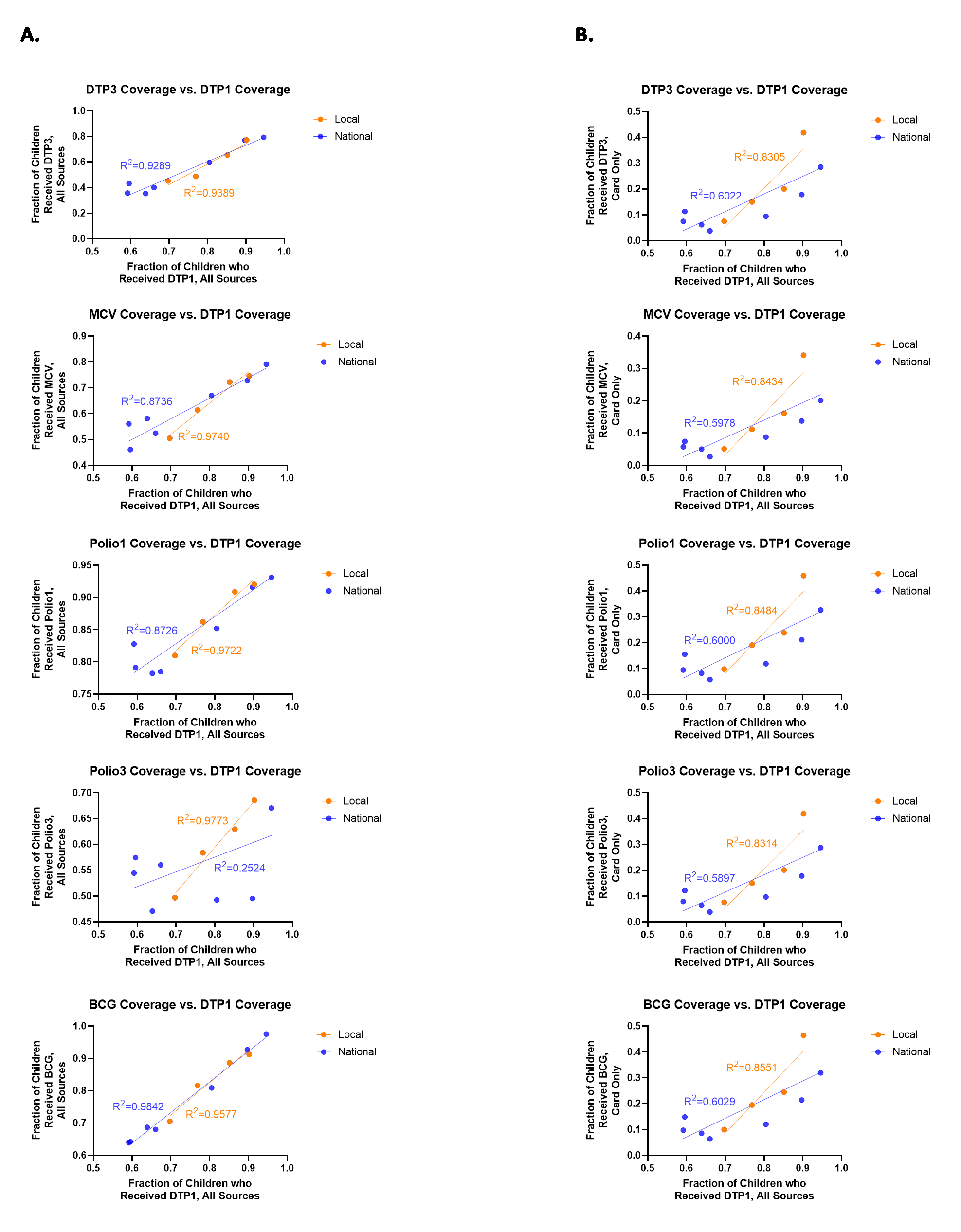

Supplement: S1 Fig — Column A shows how the inclusion of caregivers’ responses improve the linear fit compared to the relationships in column B which only considered a child vaccinated if their vaccination card contained the appropriate vaccine information. In this case, a difference was observed between the strength of the relationship observed in regions with conflict compared to the entire country. (TIF) [file pone.0281764.s001.tif]

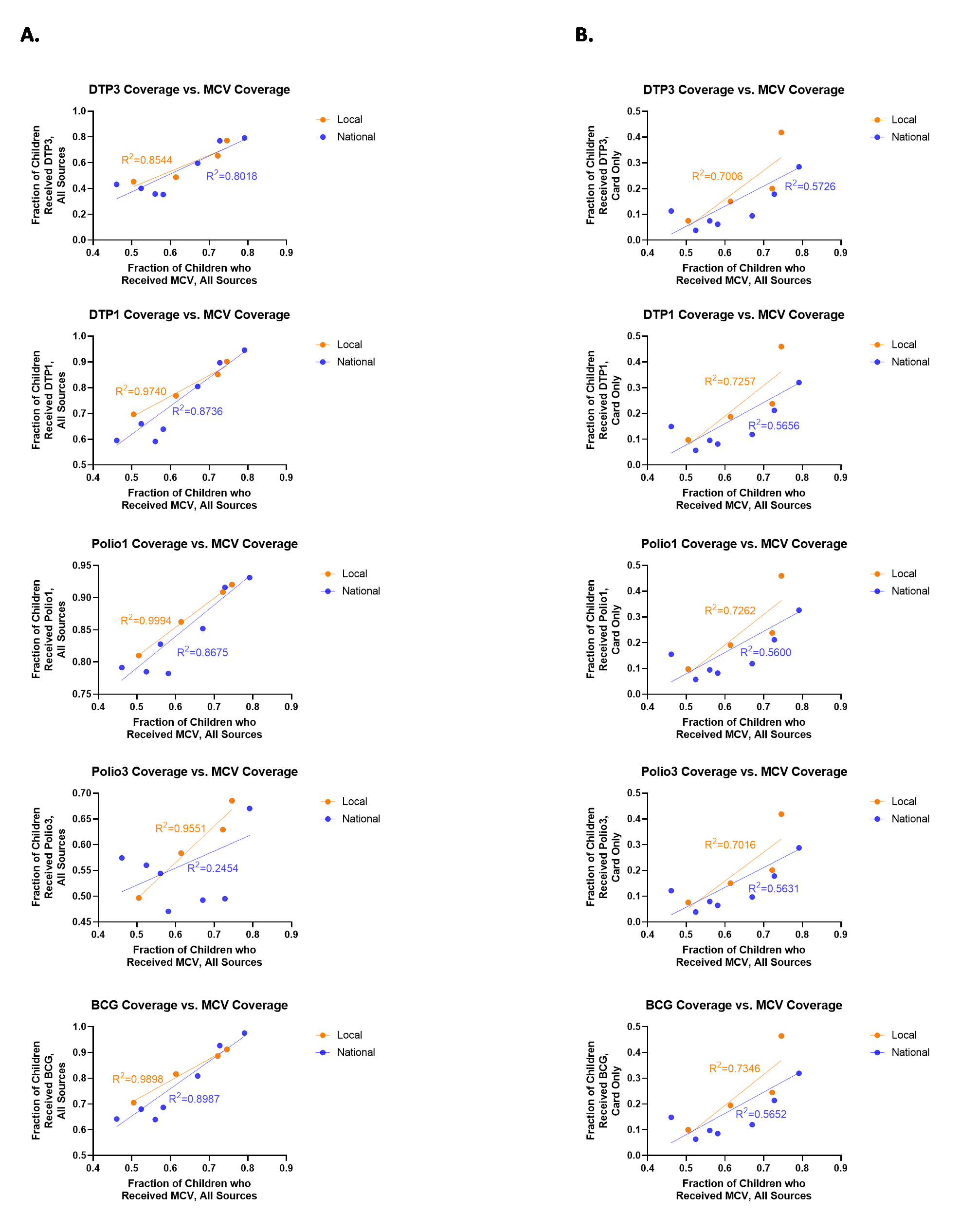

Supplement: S2 Fig — Column A shows how the inclusion of mothers’ responses improve the linear fit compared to the relationships in column B which only considered a child vaccinated if their vaccination card contained the appropriate vaccine information. In this case, a difference was observed between the strength of the relationship observed in regions with conflict compared to the entire country. (TIF) [file pone.0281764.s002.tif]

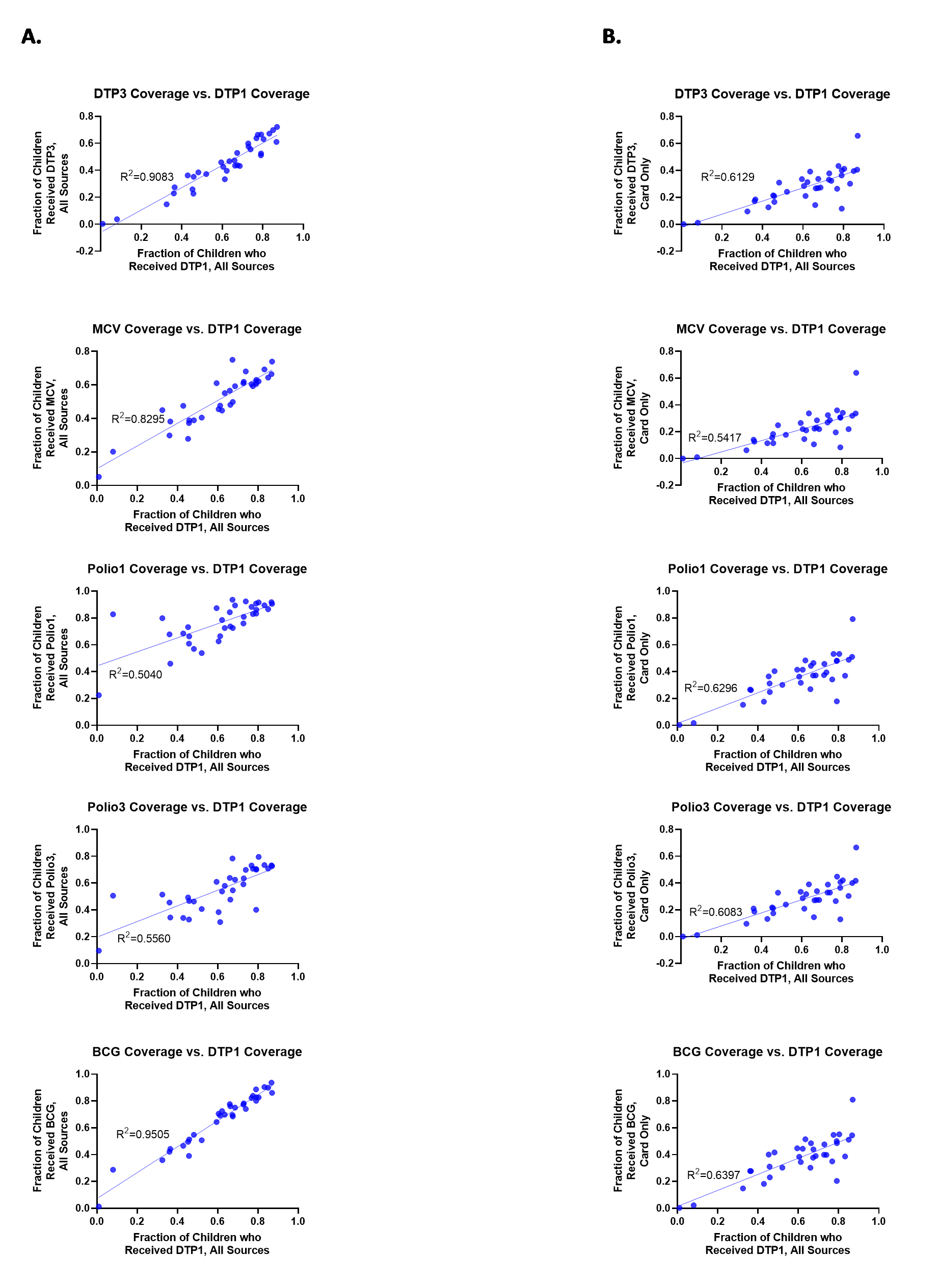

Supplement: S3 Fig — Column A shows how the inclusion of mothers’ responses improve the linear fit compared to the relationships in column B which only considered a child vaccinated if their vaccination card contained the appropriate vaccine information. This trend was not observed for either dose of the Polio vaccine, though the strengths of the fit were similar for both metrics used. (TIF) [file pone.0281764.s003.tif]

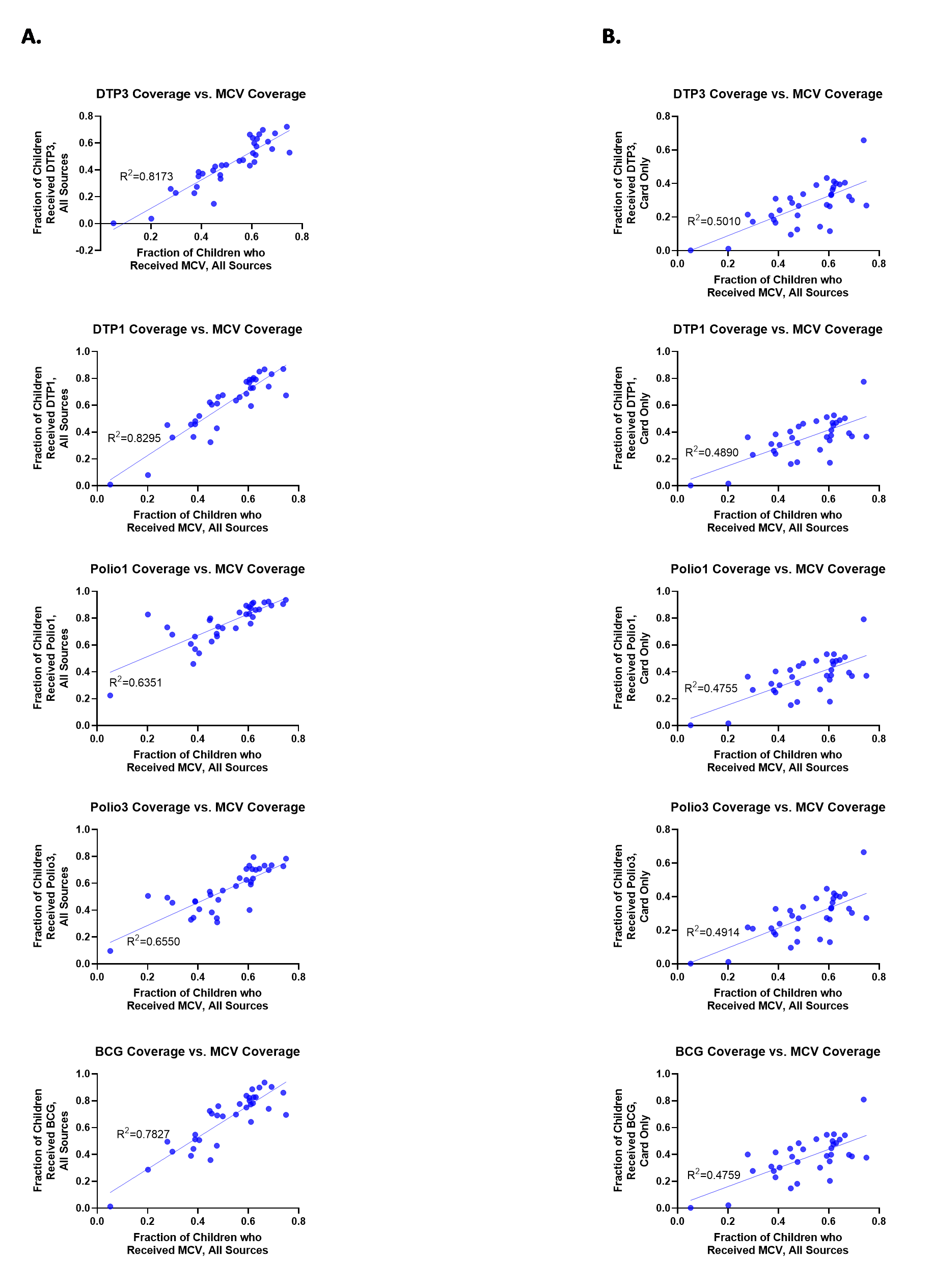

Supplement: S4 Fig — Column A shows how the inclusion of mothers’ responses improve the linear fit compared to the relationships in column B which only considered a child vaccinated if their vaccination card contained the appropriate vaccine information. Unlike for DTP1 This trend did not breakdown for either Polio vaccine dose. (TIF) [file pone.0281764.s004.tif]

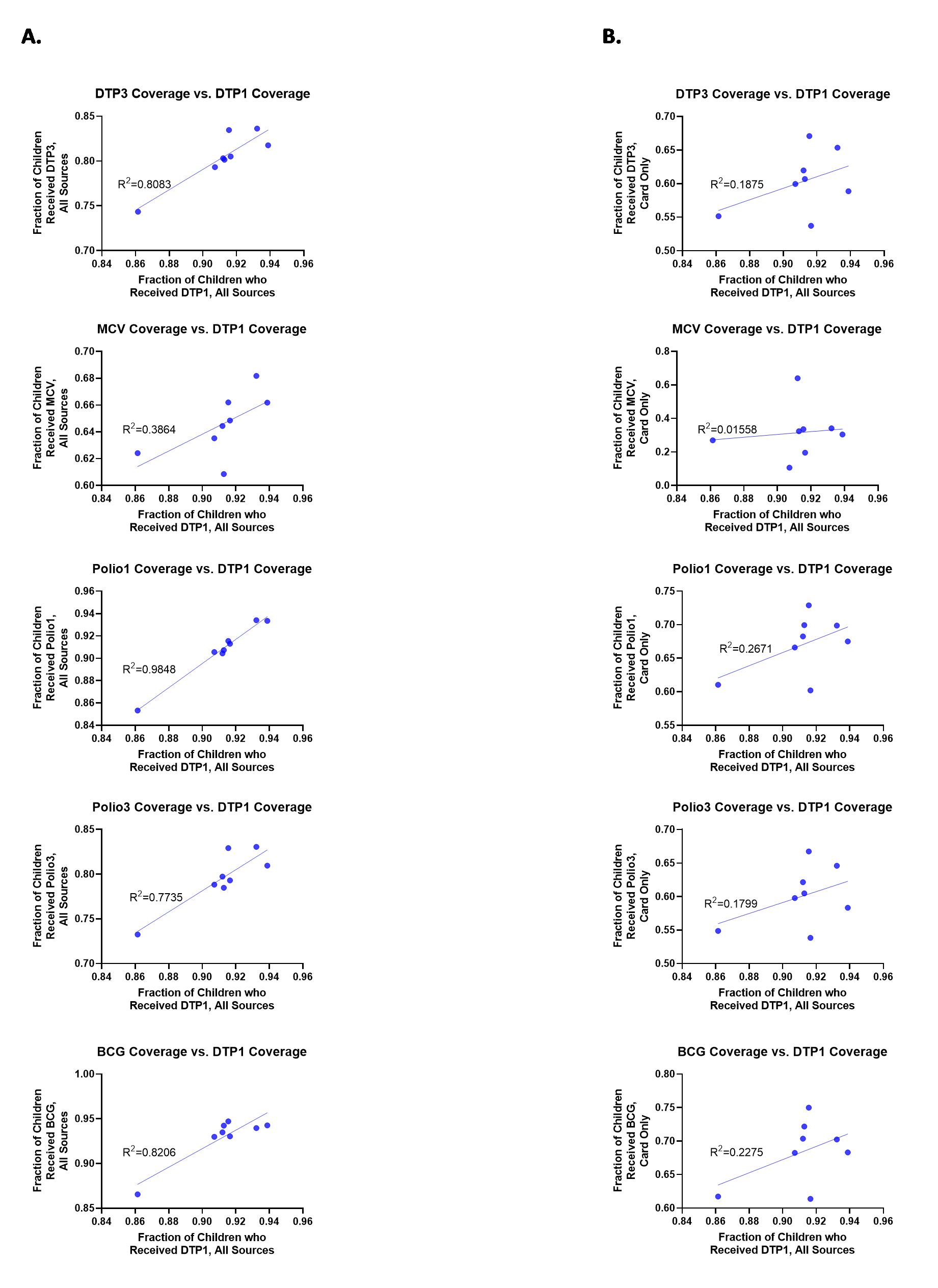

Supplement: S5 Fig — Column A shows how the inclusion of mothers’ responses improve the linear fit compared to the relationships in column B which only viewed a child as vaccinated if their vaccination card contained the appropriate information. This trend was especially apparent for DTP3, MCV, and Polio3 as the card only metric led to no linear relationship being observed, while the all sources data saw linear relationships for all cases. (TIF) [file pone.0281764.s005.tif]

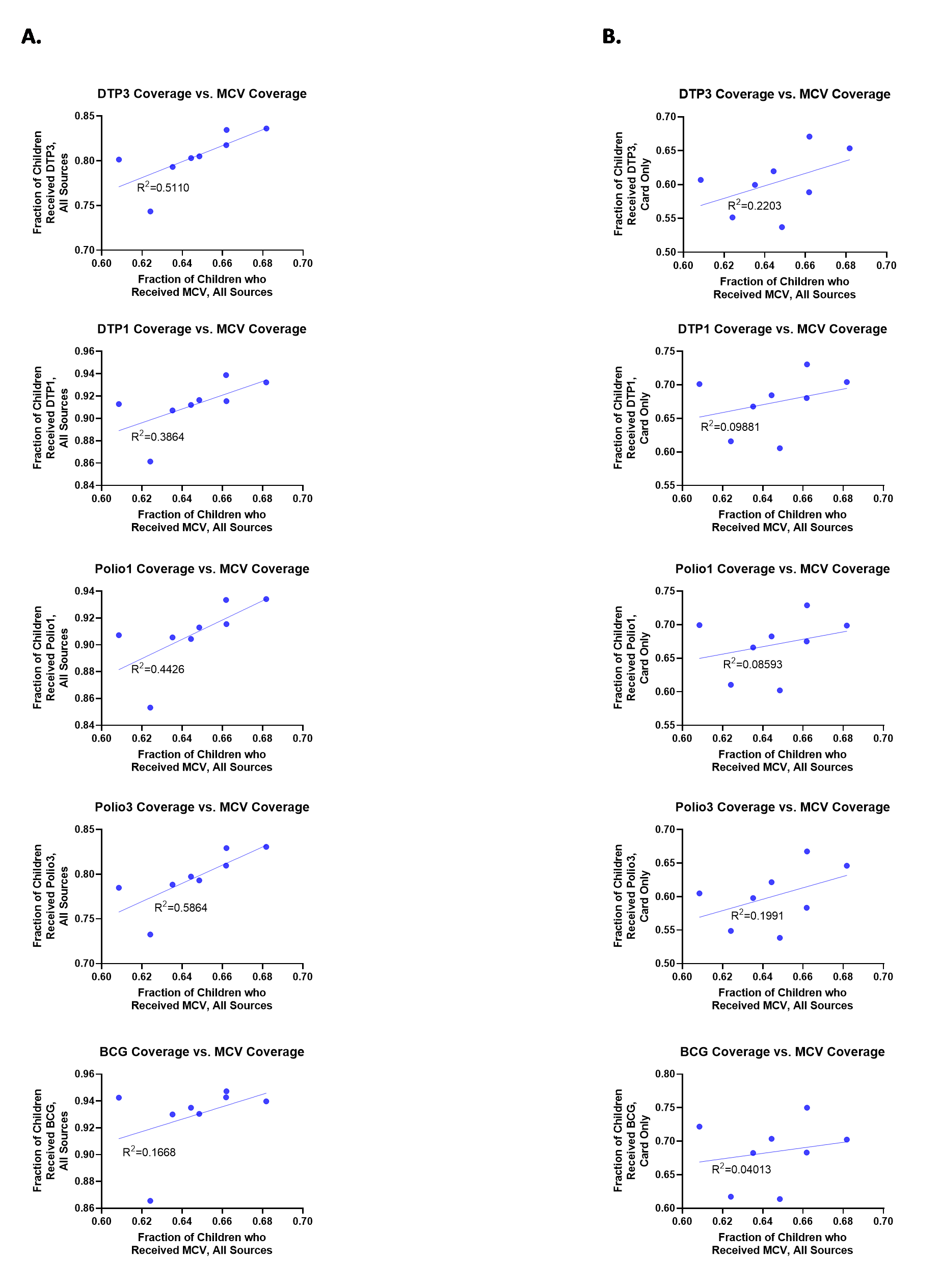

Supplement: S6 Fig — Column A shows how the inclusion of mothers’ responses improved the linear fit compared to the relationships in column B which only viewed a child as vaccinated if their vaccination card contained the appropriate information. This trend was especially apparent for DTP1, Polio1 and Polio3 as the card only metric led to no linear relationship being observed, while the all sources data saw linear associations for all of these vaccines. (TIF) [file pone.0281764.s006.tif]
