# Supplementary material for: Assessing the relationship between operationally defined zero-dose communities and access to selected primary healthcare services for children and pregnant women in emergency settings
Source: PLoS One. 2023 Feb 16;18(2):e0281764. doi: 10.1371/journal.pone.0281764 (PMC9934415; doi:10.1371/journal.pone.0281764)
Supplement: S1 Text — The accompanying supplemental contains additional statistical information for S1–S6 Figs. This is done to present the results of these supplemental figures with a degree of detail similar to the result section of the main manuscript. (DOCX) [file pone.0281764.s007.docx]

Vaccination results: DTP1 vs MCV

From our analysis, we found that all comparisons made via Chi Squared showed there was a significant association between the coverage of DTP1 and all other vaccines examined. Additional analysis also determined that there is some linear association between subnational regions’ vaccine coverage for DTP1 and the coverage of other vaccines in the area, such that as the coverage of DTP1 increases, the coverage of other vaccines also increases. In a single one-to-one comparison between the fraction of children under five who received DTP1 or the fraction of children under five who received the measles containing vaccine (MCV) and either DTP3, the first dose of the Polio vaccine (Polio1), the third dose of the Polio vaccine (Polio3), or the Bacillus Calmette–Guérin vaccine (BCG), this linear association was strongest when DTP1 was used as the independent variable. This information combined with the operational definition of zero dose communities led to the selection of DTP1 as the measurement for zero dose communities.

**DRC**

More specifically, in the Democratic Republic of Congo, a Chi Squared test was used at the national level to determine if there was a significant association between DTP1 coverage and coverage of DTP3 as well as coverage of MCV, coverage of Polio1, coverage of Polio3, and coverage of BCG. There was a statistically significant association between DTP1 coverage and DTP3 coverage (p< 0.0001), MCV coverage (p <0.0001), Polio 1 coverage (p <0.0001), Polio3 coverage (p < 0.0001) and BCG coverage (p< 0.0001). This same approach was then repeated using MCV instead of DTP with results as follows: There was a statistically significant association between MCV coverage and DTP1 coverage (p <0.0001), DTP3 coverage (p< 0.0001), Polio1 coverage (p <0.0001), Polio3 coverage (p < 0.0001) and BCG coverage (p< 0.0001). As significant association was observed in all cases, we next looked to see which vaccine proxy DTP1 or MCV produced the better linear association expected between those who are not zero dose children and vaccination status based on the value of R^2^ and checked if the slope of the line is significantly different than 0. For the *All Sources* cases, DTP1 compared to MCV generally produced a better linear fit for DTP3 (p<0.0001, R^2^ = 0.93 vs. p=0.0002, R^2^ =0.80 national; p=0.0310, R^2^ = 0.94 vs. p= 0.0756 R^2^ =0.85 local, respectively) and BCG (p<0.0001, R^2^= 0.98 vs. p<0.0001, R^2^ =0.90 national; p=0.0214, R^2^ = 0.96 vs. p= 0.0051, R^2^ =0.99 local, respectively). For Polio1 and Polio3 both proxy variables performed in a similar fashion (Polio1: p <0.0001, R^2^ = 0.87 for DTP1 vs. p<0.0001, R^2^= 0.87 for MCV at the national level, p=0.0140, R^2^ = 0.97 for DTP1 vs. p=0.0003, R^2^= 1.0 for MCV at the local level) (Polio3: p= 0.1153, R^2^= 0.25 for DTP1 vs. p=0.1213, R^2^= 0.25 for MCV at the national level, p=0.0114, R^2^ = 0.98 for DTP1 vs. p=0.0227, R^2^=0.96 for MCV at the local level). Meanwhile, for the *Card-Only Sources* cases, while both potential proxy variables performed in a fairly similar matter on the national level, on the local level DTP1 produced a stronger association compared to MCV in all cases (DTP3: p= 0.0050, R^2^ = 0.60 vs. p= 0.0070 , R^2^ = 0.57 national, p= 0.0887, R^2^ = 0.83 vs. p= 0.1630, R^2^ = 0.70 local, respectively; BCG: (p= 0.0049, R^2^= 0.60 vs. p=0.0076, R^2^ = 0.57 national, p= 0.0753, R^2^ = 0.86 vs. p=0.1429, R^2^ = 0.73, local, respectively; Polio1: p= 0.0051, R^2^ = 0.60 vs. p= 0.0081, R^2^ = 0.56 national, p= 0.0789, R^2^ = 0.85 vs. p= 0.1478, R^2^ = 0.73 local; and Polio3: p= 0.0058, R^2^= 0.59 vs. p= 0.0078, R^2^ = 0.56 national, p=0.0882, R^2^ = 0.83 vs. p=0.1624, R^2^ = 0.70 local. Thus, it was observed for nearly all cases that the linear association was both stronger and with a slope more likely to be significantly different than zero for all vaccines examined when including caregivers’ responses in the estimation of vaccination coverage (*All Sources* data) compared against the relationship observed when only using coverage information if it came from a vaccine card (*Card-Only Sources* data). The notable exception to this is in the case of Polio3 where at the national level the card only data produced a fit with an R^2^ value nearly double of the *All sources* data. Furthermore, it was observed that the relationship was at least as strong and often stronger in the specific subnational regions where conflict is more apparent compared to the relationship seen throughout all subnational regions in the country. [Figure S1] This trend was also observed with MCV. [Figure S2]

**Afghanistan**

In Afghanistan, Chi squared results were as stated: There was a statistically significant association between DTP1 coverage and DTP3 coverage (p< 0.0001), MCV coverage (p <0.0001), Polio 1 coverage (p <0.0001), Polio3 coverage (p < 0.0001) and BCG coverage (p< 0.0001). The results were similar for MCV as there was a significant association between MCV coverage and DTP1 coverage (p <0.0001), DTP3 coverage (p< 0.0001), Polio 1 coverage (p <0.0001), Polio3 coverage (p < 0.0001) and BCG coverage (p< 0.0001). Comparing the linear fits of all sources data, DTP1 resulted in a better linear fit for DTP3 coverage (DTP1 p<0.0001, R^2^ = 0.91 vs. MCV: p<0.0001, R^2^ = 0.82) and BCG coverage (DTP1: p<0.0001, R^2^ = 0.95 vs. MCV: p< 0.0001 R^2^ = 0.78). Conversely, MCV resulted in a stronger fit for both Polio1 (DTP1: p<0.0001, R^2^ = 0.50 vs. MCV: p<0.0001 R^2^ = 0.64) and Polio3 coverage (DTP1: p< 0.0001, R^2^ = 0.56 vs. MCV: p<0.0001 R^2^ = 0.66). For Card-Only sources the results were as follows: DTP3: p<0.0001, R^2^ =0.61 for DTP1; p <0.0001, R^2^ = 0.50 for MCV; BCG: p<0.0001, R^2^ =0.64 for DTP1; p<0.0001, R^2^ = 0.48 for MCV; Polio1: p<0.0001, R^2^ =0.63 for DTP1; p<0.0001, R^2^ =0.48 for MCV; Polio3: p<0.0001, R^2^ =0.61 for DTP1; p<0.0001, R^2^ =0.49 for MCV showing the improved fit when DTP1 was used compared to MCV. Again, we generally observed a stronger linear association with DTP1 when examining the *all sources* data compared against the *card-only sources* data with the exception of both doses of the Polio vaccine. [Figure S3] This trend was also maintained when MCV was used as the independent variable [ Figure S4].

**Bangladesh**

Lastly, in Bangladesh the following results were observed. There was a statistically significant association between DTP1 coverage and DTP3 coverage (p< 0.0001), MCV coverage (p <0.0001), Polio 1 coverage (p <0.0001), Polio3 coverage (p < 0.0001) and BCG coverage (p< 0.0001). The results were similar for MCV as there was a significant association between MCV coverage and DTP1 coverage (p <0.0001), DTP3 coverage (Fisher’s exact test, p< 0.0001), Polio1 coverage (Fisher’s exact test, p <0.0001), Polio3 coverage (Fisher’s exact test, p < 0.0001) and BCG coverage (Fisher’s exact test, p< 0.0001).Here, DTP1 resulted in a stronger linear association among the *All Sources* data compared to MCV for DTP3 coverage (p=0.0024, R^2^ = 0.81 vs. p = 0.0463, R^2^ =0.51, respectively) , Polio1 coverage (p<0.0001, R^2^ = 0.98 vs. p=0.0718, R^2^ =0.44, respectively) , Polio3 coverage (p=0.0040, R^2^ = 0.77 vs. p=0.0267, R^2^ =0.59, respectively) and BCG coverage (p= 0.0019, R^2^ = 0.82 vs. p= 0.3152, R^2^ =0.17, respectively).

For the Card Only sources results were similar with DTP1 performing better than MCV for DTP3 coverage (p=0.2838, R^2^ = 0.19 vs. p = 0.2406, R^2^ =0.22, respectively) , Polio1 coverage (p=0.1897, R^2^ = 0.27 vs. p=0.4810, R^2^ =0.09, respectively) , Polio3 coverage (p=0.2950, R^2^ = 0.18 vs. p=0.2677, R^2^ =0.20, respectively) and BCG coverage (p= 0.2321, R^2^ = 0.23 vs. p= 0.6343, R^2^ =0.04, respectively). As with the survey results from both DRC and Afghanistan, a stronger linear association was observed when examining the *all sources* data compared against the *card-only sources* data when either MCV was used as the independent variable [Figure S6] or DTP1 was used as the independent variable [Figure S5] though even with DTP1 as the independent variable for the Card-Only sources case regardless of R^2^ values, no slope was significant different than zero though in general the use of DTP1 resulted in all R^2^ values being increased. Especially noticeable in Figure S5 were the individual relationships between DTP1 and DTP3, MCV, and Polio3. For these three vaccines, the R^2^ values were below the cut off for a weak linear relationship to be observed when only vaccination card data was considered, but after the inclusion of responses from mothers all displayed linear relationships.

Vaccination Result: All Sources vs. Card Only

It was observed for nearly all cases in DRC that the linear association was stronger for all vaccines examined when mothers’ responses were included in the estimation of vaccination coverage (*All Sources* data) compared against the relationship observed when only using coverage information if it came from a vaccine card (Card Only data). The notable exception to this is in the case of Polio3 where at the national level the card only data produced a fit with an R^2^ value larger than the *All Sources* data. Furthermore, it was observed that the relationship was at least as strong and often stronger in the specific subnational regions where conflict is more apparent compared to the relationship seen throughout all subnational regions in the country. These trends for MCV are the same observed for DTP1. [Figure S2]. Likewise, in Afghanistan a stronger linear association was observed when examining the *All Sources* data compared against the vaccination *Card Only* data. Unlike with the DTP1 relationship, here this trend was observed for all cases [Figure S4]. As with the survey results from both DRC and Afghanistan a stronger linear association was observed when examining the *All Sources* data compared against the vaccination *Card Only* data for MCV similar to that seen for DTP1 in Bangladesh. In this case all R^2^ values were increased. Especially noticeable were the individual relationships between DTP1, Polio1 and Polio3. For these three vaccines, the R^2^ values were below the cut off for a weak linear relationship to be observed when only vaccination card data was considered, but after the inclusion of responses from mothers all displayed linear relationships. We note here that while BCG did not meet the weak linear association cut off metric, the inclusion of the mothers’ response still resulted in nearly a fourfold increase in R^2^ value.
